# High incidence of occupationnal blood exposures (OBE) in the health care workers sector of low income countries, using the example of bangui, central African Republic (CAR)

**DOI:** 10.1186/1742-4690-9-S1-P115

**Published:** 2012-05-25

**Authors:** HD Mossoro-Kpinde, CD Mossor-Kpinde, E Gbangba-Ngai, CG Kamalo

**Affiliations:** 1 Bangui, Central African Republic

## Introduction

CAR has been heavily affected by HIV (6.2%) and hepatitis B (15%) and C (3%), but has not yet developed a prevention plan against OBE, even though its health care staff, already low in numbers, is overwhelmed by a massive patient load.This study aims to assess the current OBE situation and develop a national plan for the management of these accidents.

## Methods

A preliminary cross-sector study was conducted in 2009 amongst 3 health care facilities groups in Bangui. The parameters being studied were collected using a standard form including serological status for HIV, HBV and HCV, vaccination against hepatitis B, incidents of OBE and their subsequent management.

## Outcomes

Three hundred members of the health care staff were included in the study. 9.2% had been vaccinated against hepatitis B. Thirty six percent (36%) had already been tested for HIV, with 7.3% of the tests performed within the last three months. Fifty four percent (54%) cited an incident of OBE within the last six months. Sixty eight percent (68%) of these were from accidental needle stick injuries. At the time of the accident, 39.9% knew their HIV serological status, and 22% their HBV status. Three percent had been vaccinated against hepatitis B. Three per cent (3%) of the accidents received subsequent care. The post-OBE care management did not cover hepatitis B.

## Conclusion

There is a high prevalence of OBEs in the sites studied. The number of health care staff receiving subsequent care is low. As this study was limited to Bangui, it could be interesting to conduct an exhaustive evaluation throughout CAR. Meanwhile, given the current results and the aim of safeguarding the over-stretched pool of health care staff from OBEs, efforts are required to strengthen staff capacities, manage OBEs and improve hospital hygiene in the sites studied.

